# Function of Nucleus Ventralis Posterior Lateralis Thalami in Acupoint Sensitization Phenomena

**DOI:** 10.1155/2015/516851

**Published:** 2015-06-16

**Authors:** Pei-Jing Rong, Jing-Jun Zhao, Ling-Ling Yu, Liang Li, Hui Ben, Shao-Yuan Li, Bing Zhu

**Affiliations:** ^1^Institute of Acupuncture and Moxibustion, China Academy of Chinese Medical Sciences, 16 Nanxiaojie of Dongzhimen, Beijing 100700, China; ^2^Wuhan Integrated TCM and Western Medicine Hospital, 215 Zhongshan Road, Qiaokou District, Wuhan, Hubei 43002, China

## Abstract

To observe the effect of electroacupuncture (EA) on nucleus ventralis posterior lateralis (VPL) thalami activated by visceral noxious stimulation and to explore the impact of EA on the mechanism of acupoint sensitization under a pathological state of the viscera, EA was applied at bilateral “Zusanli-Shangjuxu” acupoints. The discharge of VPL neurons was response to EA increased after colorectal distension (CRD). The stimulation at “Zusanli-Shangjuxu” acupoints enhanced discharge activity of VPL neurons under CRD-induced visceral pain. The frequency of neuronal discharge was associated with the pressure gradient of CRD which showed that visceral noxious stimulation may intensify the body's functional response to stimulation at acupoints.

## 1. Introduction

Acupoints are special locations on body surfaces where the Qi of meridians and internal organs is infused. They are also the key link underlying the interactions between meridians and internal organs. When internal organs are under a pathological state, acupoints become more sensitive [1–3]. The size and function of acupoints change accordingly with the change of visceral functions [4, 5]. Therefore in pathological conditions, the diagnostic and therapeutic effects of acupoints on visceral diseases are enhanced [6].

Spinothalamic tract is traditionally viewed as the major pathway of noxious inputs. Previous studies showed [7–10] that noxious inputs transmitted via spinothalamic tract can be affected by other noxious inputs. A current issue in neuroscience research is the mechanism underlying the peripheral and central sensitization caused by different noxious inputs [11].

This study evaluated the neuronal discharge of ventral posterior lateral nucleus (VPL; the most important brain center for somatovisceral relay) by noxious inputs from the body surface and colorectal distension (CRD). We also observed whether the effect of acupuncture on the receptive field (acupoint area) of VPL neurons on body surface was affected by visceral noxious inputs. The phenomena and mechanism of acupoint sensitization at the VPL level induced by visceral noxious inputs will be discussed.

## 2. Materials and Methods 

### 2.1. Experimental Animals

Twenty-six healthy male Sprague-Dawley rats weighing 250–300 g were provided by the Laboratory Animal Center of Academy of Military Medical Sciences (animal certificate Lot Scxr (Beijing) 2009-0017). Before experiments, the animals were fasted for 12 hours but they were not deprived of water. Throughout the experiment, the animals' body temperature was maintained between 36 and 38°C by a temperature control device (Model: CL-8; Manufacturer: RWD/China). Animal experimental methods, experimental purposes, and the disposal of animals in experiment followed the *Guidelines on Proper Care of Experimental Animals* promulgated in 2006 by the Ministry of Science and Technology.

### 2.2. Experimental Methods

#### 2.2.1. Recording Discharge of Thalamic VPL Neurons

Rats were anesthetized with an intraperitoneal injection of 10% urethane (1.0~1.2 g/kg, provided by Shanghai Sinopharm Chemical Reagent Co., Ltd.). The heads of rats were fixed on stereotaxic instruments. We incised the skin of the middle of skull and the suture was exposed by removing the resubcutaneous tissue and periosteum. Then we should adjust the frontal and back suture located in a horizontal plane. The three-dimensional location coordinates of VPL nuclei were determined according to Rat Brain Atlas [12] 3.0~4.0 mm behind the anterior fontanel, 3.0~3.5 mm next to skull sutures. Under observation with a surgical microscope, the tip of the glass microelectrode was inserted to VPL nuclei through the skull hole by the microelectrode manipulator (5000~5800 *μ*m beneath the surface of the brain). Impedance at the tip of the glass microelectrode was set at 10–15 MΩ (filled with 2% pontamine sky blue). When the target neurons were identified, 2% agar was perfused onto the skull surface to protect the brain tissue from drying and reduce volatility caused by breathing.

For all recorded neurons, the responses to mechanical stimulations applied to their peripheral receptive field were checked to identify the distribution and size of the receptive fields (mechanical stimulations include touch and pressure by von Frey hairs (von Frey Model 2390; U.S. IITC Company), skin stimulation by tooth tweezers, and acupuncture stimulation). We also observed responses of these neurons to CRD. Only neurons that responded to both the mechanical stimulation on the skin receptive field and the 10 mmHg of CRD were included as the objects of observation (and were named as convergent neurons or CN).

#### 2.2.2. Colorectal Distension

**Figure 1:**
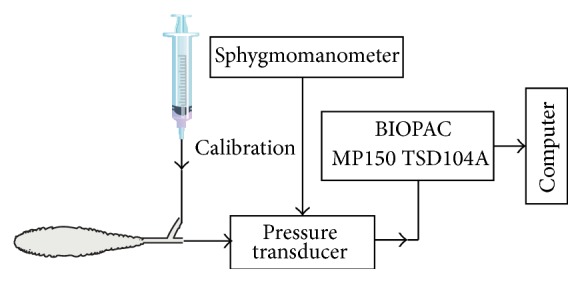
Experimental layout of colorectal distension.

A 4 to 6 cm long balloon was made from a disposable condom tip and tied on a 4 mm diameter hose (Figure 1; BIOPAC Amplifier Module Model: MP150 System TSD104A; Manufacturer: BIOPAC Company, USA). The balloon was inserted through the anal orifice straight into the colon. The depth was approximately 4 cm. Three to five drops of the warmed paraffin oil were smeared on the balloon's surface before the balloon was placed into the colon to avoid direct damage to the inner wall of the colon and anus. The distance from balloon end to anus was about 0.5 cm. 20–80 mmHg CRD stimulus was given via a syringe, with the duration of about 30 s. The activation of convergent neurons was observed at different intensities of CRD stimulation. Pressure ≥ 40 mmHg was identified as visceral noxious stimulation [13]. The time interval between CRD stimulations was no less than 10 min to avoid colorectal sensitization caused by hyper stimulation.

#### 2.2.3. EA

EA was applied at bilateral “Zusanli-Shangjuxu” points. The stimulation was set as a square wave pulse with a width of 5 ms and frequency of 20 Hz. The intensity was 1.5 times of the threshold of A*δ* fiber [14] (the average threshold intensity of A*δ* fiber reflex was 1.54 ± 0.50 mA) and the time for EA was 30 s. The discharge of VPL neurons to EA was observed before and after CRD.

### 2.3. Experimental Procedure

**Figure 2:**
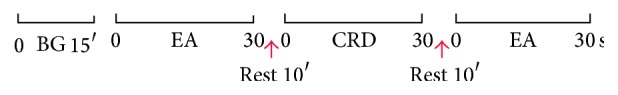
Experimental flow chart.

(1) The background discharge of convergent neurons was recorded for 10–15 min using microelectrode amplifier (Model: MEZ 8201; Manufacturer: Nihon Kohden) and biological signal acquisition and analysis system (Model: MICRO 1401; Manufacturer: British CED Company). (2) EA was applied at bilateral “Zusanli-Shangjuxu” points for 30 seconds. (3) After an interval of 10 minutes, different intensities of CRD were given to rats for 30 seconds. The discharge of the convergent neurons to nonnoxious stimuli (20 mmHg), noxious stimuli (40 mmHg), and strongly noxious stimuli (60, 80 mmHg) was recorded, respectively, to observe the activation of convergent neurons by different intensities of CRD. (4) After an interval of 10 minutes, EA was once again applied at bilateral “Zusanli-Shangjuxu” points for 30 seconds. The discharge of VPL neurons in response to EA stimulation before and after different intensities of CDR was observed to test the dose-effect relationship between stimulus intensity and response (Figure 2).

### 2.4. Statistical Analysis

The data was analyzed with Spike-II (the data analysis software of MICRO 1401 biological signal acquisition and analysis system) and SPSS 13.0 software. The number of neuronal discharge of VPL neurons in every 30 seconds and the activation/inhibition rate were counted and the mean and variance of neuronal discharge before and after the EA intervention were calculated. Comparison between groups was made with independent sample *t*-test. *P* < 0.05 was considered as statistically significant.

### 2.5. Histological Localization

**Figure 3:**
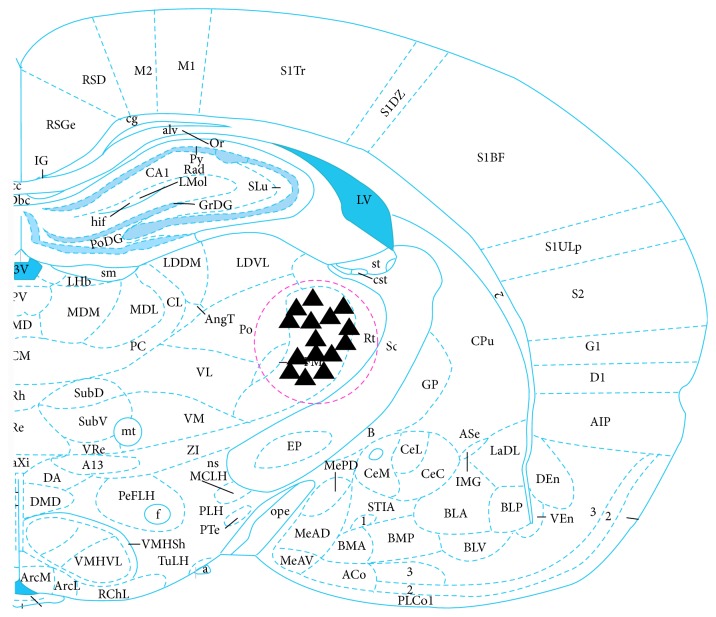
▲ refers to the location of VPL neurons.

When recording of neuronal discharge was completed, 20 *μ*A of negative direct current was passed to the glass microelectrode via the microelectrode amplifier for 20–30 min. Pontamine sky blue in the glass microelectrode was imported into VPL nuclei to mark the position of recording electrode. Thereafter, the rats were euthanized and perfused through the heart with 4% of paraformaldehyde. Then the rats' brains were removed and fixed. After an interval of 72 hours, frozen sections of the brain were cut for H&E staining (Figure 3). Recording points that were not located in the VPL nuclei were removed from the study.

## 3. Results

### 3.1. General Characteristics of the Responses of VPL Neurons

**Figure 4:**
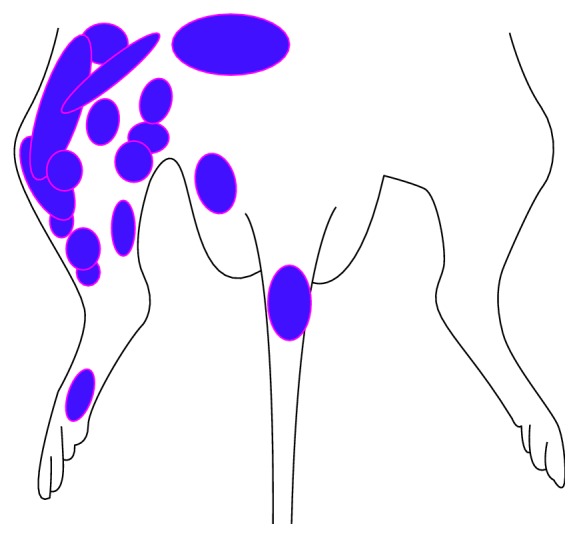
Distribution of the receptive fields of partial VPL neurons.

A total of 126 VPL neurons that responded to mechanical stimulations from the body surface were identified in the 26 male SD rats, by referring to the Brain Atlas of the rat [14]. Figure 3 illustrates part of the pontamine sky blue positioning of VPL neurons. Their receptive fields were distributed at the poster lateral of the contralateral body, tail, hips, or hind legs. The receptive fields of most neurons were small but had clear boundaries. The receptive fields could be activated by gentle brushing or tapping by von Frey filaments (Figure 4).

### 3.2. The Influence of Different Intensities of CRD on the Discharge of VPL Neurons

**Figure 5:**
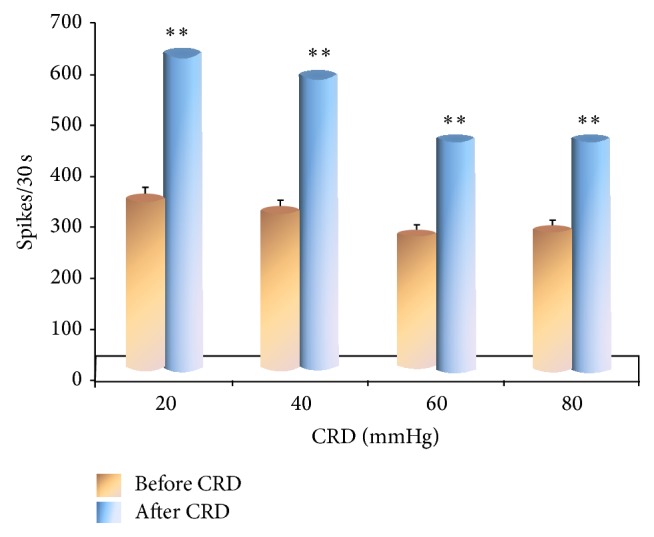
Discharge of VPL neurons before and after CRD.

We isolated 54 convergent neurons from the 126 VPL neurons that responded to inputs of mechanical stimulation and systematically observed the discharge of 9 of the 54 convergent neurons caused by different intensities of CRD stimulation. The results showed that, after CRD stimulation ranging from 20 to 80 mmHg, the discharge frequency of VPL neurons significantly increased in rats more than before CRD stimulation (*P* < 0.01) (Figure 5).

### 3.3. The Influence of Different Intensities of CRD on the Discharge Frequency of VPL Neurons Induced by EA

The discharge of 45 convergent neurons was observed when rats were given different intensities of CRD. Among them, 12 convergent neurons were chosen from rats receiving 20 mmHg of nonnoxious CRD, 11 from rats receiving 40 mmHg of noxious CRD, 12 from rats receiving 60 mmHg of strong nociceptive CRD, and 10 from rats that were given 80 mmHg of strong nociceptive CRD.

**Figure 6:**
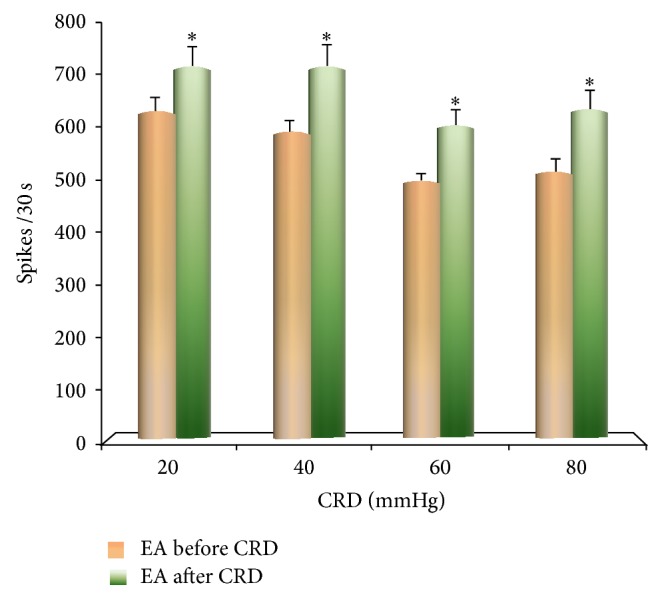
The influence of EA on the discharge of VPL neurons before and after CRD.

Equal intensities of EA were given to rats for 30 seconds before and after CRD. The results showed that the discharge frequency of VPL convergent neurons induced by EA increased significantly after CRD more than before CRD when rats were given different intensities of CRD (*P* < 0.05) (Figure 6).

### 3.4. The Influence of Different Intensities of CRD on the Discharge Number of VPL Neurons Induced by EA

After rats were given CRD, the discharge from VPL convergence neurons induced by EA increased over the discharge before CRD: 20 mmHg–15.38% ± 8.27; 40 mmHg–25.22% + 7.80; 60 mmHg–36.28 + 8. 18; 80 mmHg–38.40 + 8.32. Differences were statistically significant (*P* < 0.05).

**Figure 7:**
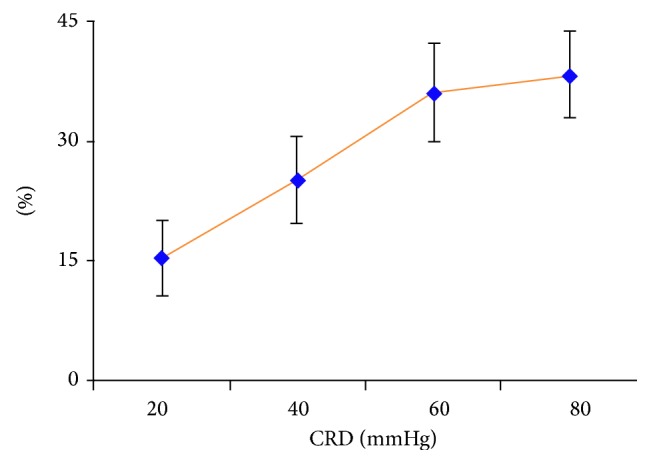
The influence of different intensities of CRD on VPL neuronal discharge induced by EA.

As the intensity of CRD stimulation increased, there also was an increase in the percentage of the discharge number of VPL neurons from EA at acupoints. A certain dose-effect relationship could be observed between stimulation and response. It showed that acupoints on the body surface were sensitized after CRD. The effect of EA on acupoints was enhanced. The sensitization of acupoints increased as the intensity of visceral noxious stimulation increased (Figure 7).

The above results showed that noxious visceral stimulation facilitated the responses of VPL neurons to inputs of EA stimulation from acupoints on body surface.

## 4. Discussion

Our previous studies have shown that most neurons which responded to somatic afferent inputs also responded to inputs from CRD or skin vibrotactile stimulation. In most cases, the response was shown as sensitization of neurons. Responses of more than 50% of neurons to skin vibrotactile stimulation could be enhanced by CRD previously applied to experimental animals [15].

Results of this study showed that, within a certain intensity range, the discharge frequency of VPL convergent neurons increased as the intensity of CRD stimulation increased. Since CRD had an activation effect on spinal cord neurons, when EA was applied at acupoints after CRD, the discharge of VPL convergent neurons had a significant increase more than before CRD. This confirms that noxious visceral distension can sensitize VPL neurons making them respond more strongly to inputs from EA applied to acupoints on skin receptive fields. In other words, the neural facilitation of VPL neurons after noxious visceral stimulation led to dynamic changes of the response from the acupoint sensitized. As the intensity of visceral noxious stimulation increased, its effect on sensitization of acupoints on body surface also strengthened and showed a clear dose-response relationship. Our results show that VPL neurons are involved in the dynamic process of acupoint sensitization.

The thalamus is the most important brain structure to relay somatic and visceral afferent inputs to the cerebral cortex. There are three projection systems from the spinal cord to the bottom part of ventral thalamus: the spinothalamic tract, the cervical spinal column, and the postsynaptic dorsal column ascending fibers. A study by Yang et al. [16] on VPL of rats showed that 94% of VPL neurons could be activated by nonnoxious and noxious stimuli applied at peripheral receptive fields, whereas 6% of VPL neurons only responded to noxious stimulation. No VPL neurons responded only to nonnoxious stimulation. Nearly 60% of VPL neurons also responded to CRD, primarily with activation. VPL neurons, therefore, are involved not only in the transmission and processing of somatic sensory inputs, but also in the transmission and processing of visceral nociceptive inputs.

We observed in the rat thalamus VPL experiment that most neurons that responded to haptic inputs from contralateral body also responded to CRD and skin vibration tactile stimuli. The responses of more than half of the neurons to skin vibrotactile stimulation could be enhanced by CRD conditioned stimulation applied previously. In contrast, the responses of VPL neurons to CRD were not enhanced by skin tactile stimulation, if the order of conditioned stimulation was reversed; that is, the skin stimulation was given before CRD. Moreover, the effect was mainly shown as an inhibitory effect. A possible explanation for acupoint sensitization is that repeated CRD may cause the irritability of intestinal wall, which can be viewed as one type of visceral inflammation, and induces sensitization of afferent neurons [17]. Visceral noxious stimulation could also significantly enhance neuronal responses to skin tactile stimulation. The enhancement effect may be related with hyperalgesia caused by visceral disease [18, 19].

Many previous studies suggest that only noxious stimulation can significantly inhibit the afferent transmission of nociceptive inputs [18]. However, we observed that, at the single cell level, a gentle touch on skin could produce inhibitory effect on hypothalamic neurons' response to CRD, though this inhibitory effect was usually mild and transient. The conditioned stimulation of CRD significantly improved the responses of thalamic neurons to tactile inputs. The facilitation effect was related with the activity of excitatory intermediate neurons. That is, excitatory intermediate neurons could enhance the after-effects of excitatory responses caused by CRD and prolong the discharge duration of VPL neurons. If skin tactile stimulation was given after CRD, the discharge number of VPL neurons was higher than the discharge number when only CRD or skin tactile stimulation was given. The excessive sensitivity of central neurons to skin tactile stimulation may be related with hyperalgesia [18, 19]. Though only a few such sensitive points were found on skin receptive fields, the sensitization effect was lasting and was longer than the effect directly caused by skin stimulation. The effect also lasted significantly longer than the inhibitory effect of tactile stimulation on CRD response [17]. In this case, visceral nociceptive inputs had a stronger effect on the tactile inputs than the other way around, at least at the single cell level of thalamus VPL neurons. However, it should be emphasized that the perception of the visceral pain depends on the group response of neurons, which includes the interaction and feedback among nerve centers at cerebral cortex, thalamus, and other areas.

Our study showed that nociceptive stimulation of CRD could make VPL neurons more sensitive to EA stimulation applied at skin receptive fields. It indicates that viscera pathological condition can facilitate the afferent inputs from stimulation at the body surface. The interaction between somatic and visceral inputs occurs at the lumbosacral segments of the spinal cord. The segments (L1–L3) not only integrate information from the skin on lower abdomen and hind legs, but also are the location of afferent neurons for “Zusanli-Shangjuxu” points which were elected in our experiment and dominate the lower digestive tract. Many sensitive points on body surface are distributed at relevant acupoint zones that have a regulatory effect on digestive system functions. The phenomenon that visceral nociceptive inputs can facilitate the neural responses to afferent inputs from the body surface at corresponding spinal segments may be related with the mechanism underlying referred pain. It also provides a scientific explanation for the Chinese medical theory of “pain as acupoints” and “essence of acupuncture points.”
